# Resilience, renewal and hope in Australian Indigenous-led primary health care initiatives

**DOI:** 10.1017/S1463423621000712

**Published:** 2021-11-12

**Authors:** Rhonda Marriott, Tracy Reibel

**Affiliations:** 1 Ngangk Yira Research Centre for Aboriginal Health and Social Equity, Murdoch University; 2 Telethon Kids Institute, University of Western Australia

Aboriginal and Torres Strait Islander cultures in Australia are the oldest continuous cultures globally, established over 60 000 years. The fact that Australian Indigenous peoples have sustained their practices, languages and societies over such a phenomenally long period of time is evidence of strengths beyond our contemporary imagining.

Dispossession of Indigenous peoples’ lands across the globe promulgated by the enlightenment has had severe impacts (Murray, 2007; Imbruglia, 2015; Kolinjivadi, 2018). With increased populations and industrialisation over the last two centuries, clean rivers and air and pristine lands have been threatened, further depleting the life forces of Indigenous cultures.

Twenty-first century populations everywhere are now engaged in global struggles: climate changes impacting farming production; environmental degradation stemming from monocultural agricultural systems, mining and excessive use of fossil fuels; and a plethora of disadvantageous social conditions including wars leading to societal destabilisation, the ever present and widening poor/rich divide and the current COVID-19 pandemic. Despite the pandemic, Australia still has a robust economy. Even so, Australian Indigenous peoples continue to disproportionately experience far poorer health outcomes than the non-Indigenous population (AIHW, 2018); interrupting their opportunity to fully participate in education and employment.

In the Uluru Statement from the Heart (2017), Australian Indigenous peoples say: “*the ancestral tie between the land, or ‘mother nature’, and the Aboriginal and Torres Strait Islander peoples who were born therefrom, remain attached thereto, and must one day return thither to be united with our ancestors*”. In asserting the sacred link to the land, there is spirit and generosity in also stating that “*with substantive constitutional change and structural reform, we believe this ancient sovereignty can shine through as a fuller expression of Australia’s nationhood*”.

As well as the Uluru Statement, Australian Indigenous peoples are carefully choosing their ‘battles’ to rearticulate their cultural knowledges, both retaining and translating ancient knowledges into the present. This includes thoughtfully designing approaches to address a range of imposed disadvantage – in health, employment, education and environmental management – while continuing to practice their culture everyday and living on country.

An atmosphere of resilience, renewal and hope is propelling Australian Indigenous people to lead research and development in primary health care to address entrenched, some might say ‘wicked’, problems. This work though is not about the unsolvable. Rather it is about using deeply ingrained knowledge systems, passed down through countless generations melding these with contemporary Indigenous knowledge formulated in science alongside Indigenous ways of knowing, being and doing (Martin and Mirraboopa, 2003; Nicholas, 2018) for the benefit of diverse communities.

The persistent call by Australian Indigenous peoples for local solutions to meet local needs incorporates consistent elements due to similar foundations of need. In the report ‘In good hands: the people and communities behind Aboriginal-led solutions’, Josie Douglas’s foreword notes that: “*sovereignty is about land and more —it’s also about local government, self-government and control over service delivery…One of the best ways to achieve this end is through the vast network of community-controlled organisations that serve the needs of communities across the country”* (2019 p. 5).

This special issue collection presents Australian Indigenous-led primary health care research and development papers. When we invited colleagues across Australia to contribute to this collection, we knew the papers would be diverse, and we were not disappointed. This collection showcases approaches that reflect a life course approach to solutions which will support improvements in Australian Indigenous health outcomes.

From a short report on the role of Aboriginal leadership in community health programs (Stroud and colleagues), to parents’ views of trauma recovery in Chamberlain and colleagues ‘Healing the Past by Nurturing the Future’ and community-controlled aged care (Dawson and colleagues), this collection considers: building workforce leadership capacity and capability; social and emotional wellbeing; safe infant sleeping practice and growing strong brains; primary health care for homeless families; cultural security in mental health and rehabilitation settings; aged care principles, practices and actions; decolonising methodologies and, the potential role of community pharmacists in ear health.

With a mix of research and development papers, the strength of this collection lies in the multidisciplinary teams of Indigenous and non-Indigenous researchers working in academic, government and non-government settings and using knowledge and evidence created by and with Indigenous peoples to address areas of primary health care that remain underdeveloped in mainstream services, or where services have been missing in action for Aboriginal and Torres Strait Islander communities. The papers work from strengths-based perspectives, acknowledging the profoundly wholistic ‘mind, body, spirit’ cultural positioning that Australian Indigenous peoples maintain. This perspective can only really be understood when Indigenous people develop, lead, coordinate and research the very best practices which emanate from Indigenous lenses being cast across everyday issues of primary health concern. Maternal and infant health, growing strong children, addressing mental health concerns and caring for elders through knowing, being and doing in culturally safe and secure ways.

We commend this collection to anyone with an interest in primary health care. How best to provide health care services can be accomplished differently to suit the requirements of communities, promoting self-determination when Australian Indigenous peoples lead the solutions. Aboriginal and Torres Strait Islander communities have the solutions. What is needed now are for governments to listen to the knowledge, wisdom and expertise of community leaders and initiate flexible funding arrangements which support local solutions to address local primary health care needs. We will then have meaningful equity and equality in the delivery of primary health services. This is not a utopian concept; it is how primary health care should be.
